# Challenges and opportunities associated with the MD Anderson IMPACT2 randomized study in precision oncology

**DOI:** 10.1038/s41698-022-00317-0

**Published:** 2022-10-27

**Authors:** Henry Hiep Vo, Siqing Fu, David S. Hong, Daniel D. Karp, Sarina Piha-Paul, Vivek Subbiah, Filip Janku, Aung Naing, Timothy A. Yap, Jordi Rodon, Jaffer A. Ajani, Carrie Cartwright, Amber Johnson, I-Wen Song, Jennifer Beck, Michael Kahle, Graciela M. Nogueras-Gonzalez, Vincent Miller, Calvin Chao, David J. Vining, Donald A. Berry, Funda Meric-Bernstam, Apostolia-Maria Tsimberidou

**Affiliations:** 1 Department of Investigational Cancer Therapeutics, The University of Texas MD Anderson Cancer Center, 1515 Holcombe Blvd., Houston, TX 77030 USA; 2 Department of Gastrointestinal Medical Oncology, Division of Cancer Medicine, The University of Texas MD Anderson Cancer Center, 1515 Holcombe Blvd., Houston, TX 77030 USA; 3 Khalifa Institute for Personalized Cancer Therapy, The University of Texas MD Anderson Cancer Center, 1515 Holcombe Blvd., Houston, TX 77030 USA; 4 Department of Biostatistics, The University of Texas MD Anderson Cancer Center, 1515 Holcombe Blvd., Houston, TX 77030 USA; 5 Foundation Medicine, Inc., Cambridge, MA USA; 6 Tempus, Chicago, IL USA; 7 Department of Abdominal Imaging, The University of Texas MD Anderson Cancer Center, 1515 Holcombe Blvd., Houston, TX 77030 USA

**Keywords:** Drug development, Cancer genomics, Cancer therapy

## Abstract

We investigated the challenges of conducting IMPACT2, an ongoing randomized study that evaluates molecular testing and targeted therapy (ClinicalTrials.gov: NCT02152254). Patients with metastatic cancer underwent tumor profiling and were randomized between the two arms when eligibility criteria were met (Part A). In Part B, patients who declined randomization could choose the study arm. In Part A, 69 (21.8%) of 317 patients were randomized; 78.2% were not randomized because of non-targetable alterations (39.8%), unavailability of clinical trial (21.8%), other reasons (12.6%), or availability of US Food and Drug Administration (FDA)-approved drugs for the indication (4.1%). In Part B, 32 (20.4%) of 157 patients were offered randomization; 16 accepted and 16 selected their treatment arm; 79.0% were not randomized (patient’s/physician’s choice, 29.3%; treatment selection prior to genomic reports, 16.6%; worsening performance status/death, 12.7%; unavailability of clinical trials, 6.4%; other, 6.4%; non-targetable alterations, 5.7%; or availability of FDA-approved drugs for the indication, 1.9%). In conclusion, although randomized controlled trials have been considered the gold standard for drug development, the execution of randomized trials in precision oncology in the advanced metastatic setting is complicated. We encountered various challenges conducting the IMPACT2 study, a large precision oncology trial in patients with diverse solid tumor types. The adaptive design of IMPACT2 enables patient randomization despite the continual FDA approval of targeted therapies, the evolving tumor biomarker landscape, and the plethora of investigational drugs. Outcomes for randomized patients are awaited.

## Introduction

In 2007, it was believed that targeted therapy against tumor alterations would not be effective for patients with solid tumors owing to the rarity of individual molecular alterations and limited number of targeted drugs. In contrast, the field of hematologic malignancies had already advanced to treat patients with acute or chronic leukemias on the basis of their molecular profiles, and tissue biopsy and blood analysis were routinely used for diagnostic purposes and for monitoring residual disease or emergence of resistance to treatment. In 2001, imatinib was approved by the US Food and Drug Administration (FDA) because it significantly improved overall survival of patients with newly diagnosed chronic myeloid leukemia by targeting *BCR-ABL*, which is expressed in 95% of patients with this disease^1^. Despite the prevailing wisdom, in 2007, we started the Initiative for Molecular Profiling and Advanced Cancer Therapy (IMPACT) study in the Department of Investigational Cancer Therapeutics at The University of Texas MD Anderson Cancer Center, a study which included early-phase clinical trials across tumor types. Until the initiation of IMPACT, the selection of patients for phase I clinical trials with novel agents was based solely on clinical trial availability. With IMPACT, patients with advanced cancer underwent tumor molecular testing and were matched to phase I clinical trials of targeted treatments.

In our first IMPACT study, treatment assignment (targeted vs. non-targeted) was determined after review of clinical, laboratory, pathologic, and tissue genomic data (Clinical Laboratory Improvement Amendments [CLIA]-certified laboratory). Patients whose tumors had an actionable molecular aberration were preferably treated in a clinical trial with matched targeted therapy (MTT), when available^2^. If MTT was unavailable, patients were treated with non-matched therapy (non-MTT). A patient’s tumor was considered matched with a targeted therapy if the drug was known to inhibit the aberration at low nanomolar concentrations or if an antibody targeted the alteration product^3^. Of 1144 sequentially analyzed patients, 460 (40.2%) had ≥1 molecular aberration (including actionable and nonactionable). In patients with 1 aberration, MTT (*n* = 175) compared with non-MTT (*n* = 116) was associated with a higher overall response rate (ORR; 27% vs. 5%; *P* < 0.0001), longer time to treatment failure (TTF; median, 5.2 vs. 2.2 months; *P* < 0.0001), and longer overall survival (OS; median, 13.4 vs. 9.0 months; *P* = 0.017)^2^. MTT was also associated with longer TTF compared with the patients’ TTF with prior systemic therapy (paired analysis, 5.2 vs. 3.1 months, respectively; *P* < 0.0001) and was an independent factor predicting response (*P* = 0.001) and TTF (*P* = 0.0001) in multivariate analysis^2^.

Despite these encouraging results, there were several limitations to the IMPACT study. Multiple molecular alterations and MTT agents were included. The study was not randomized, and, therefore, unknown confounding factors may have contributed to higher rates of response and longer TTF and survival in patients with molecular alterations treated with MTT compared to those treated with non-MTT. Moreover, since some patients received >1 agent, such as MTT combined with cytotoxic agents, some responses might not have been due to the MTT. It is also plausible that patients treated with MTT had a more favorable prognosis than the unmatched patients by virtue of their tumors having the target and that treatment outcomes were confounded by the disease’s natural history.

To address the limitations of the first IMPACT study (Supplemental Information), in 2014, we initiated IMPACT2, a phase 2 randomized trial with an adaptive innovative study design. Evidence from randomized controlled trials (RCTs) is the gold standard of medical research, and FDA approval of investigational agents is often based on evidence originating from well-conducted RCTs^4^. These trials offer a robust comparative and quantitative design to assess the cause-and-effect relationship between an intervention and an endpoint, providing high-quality evidence to assess the effectiveness and safety of an intervention^5^. The objective of IMPACT2 is to determine whether patients treated on the basis of tumor genomic alterations have longer progression-free survival (PFS) than those whose treatment is not selected on the basis of genomic alterations. Despite being the gold standard in drug development, randomized, controlled trials are arduous. IMPACT2 began with a 1:1 randomization design (Part A) between two arms (MTT vs. non-MTT). Taking into consideration evolving data in precision oncology and the wish to incorporate patient preference into treatment selection, the trial was amended in March 2019 to include Part B, a “patient-preference” cohort where patients eligible for randomization are now offered randomization between the two arms or selection of their preferred cohort. Herein, we present the challenges and opportunities associated with patient randomization in IMPACT2, along with the current status and baseline characteristics of patients enrolled on the study.

## Results

### Patients

As of October 7, 2021, in total, 600 patients (391 from Part A and 209 from Part B) were enrolled on the study and 85 had been randomized. Patient baseline characteristics are summarized in Table 1A. The median age was 59.4 years (range, 18.8–84.3); 51.5% were women. The median number of prior therapies was three (range, 0–17); ECOG performance status was 0 in 80 (13.3%) patients and 1 in 520 (86.7%) patients. The median number of metastatic sites was 2 (range, 0–12). Overall, 361 (60.2%) patients had high lactate dehydrogenase (LDH) levels (≥ULN), and 54 (9.0%) patients had low albumin levels (<lower limit of normal).

**Table 1:** A. Patient baseline characteristics. B Genomic alterations of patients with ≥1 targetable alteration*.

TABLE 1A. Patient Baseline Characteristics	Group	No. of patients, Part A (%)	No. of patients, Part B (%)	Total no. of patients (%)
		(*N* = 391)	(*N* = 209)	(*N* = 600)
Age, years	<60	194 (49.6)	118 (56.5)	312 (52.0)
	≥60	197 (50.4)	91 (43.5)	288 (48.0)
Gender	Female	197 (50.4)	112 (53.6)	309 (51.5)
	Male	194 (49.6)	97 (46.4)	291 (48.5)
No. of prior therapies	≤3	234 (59.8)	84 (40.2)	318 (53.0)
	>3	151 (38.6)	125 (59.8)	276 (46.0)
	UNK	6 (1.5)	0	6 (1.0)
PS	0	50 (12.8)	30 (14.4)	80 (13.3)
	1	341 (87.2)	179 (85.6)	520 (86.7)
No. of metastatic sites	0–2	234 (59.8)	129 (61.7)	363 (60.5)
	>2	157 (40.2)	79 (37.8)	236 (39.3)
	UNK	0	1 (0.5)	1 (0.2)
Liver metastases	No	236 (60.4)	113 (54.1)	349 (58.2)
	Yes	155 (39.6)	95 (45.5)	250 (41.7)
	UNK	0	1 (0.5)	1 (0.2)
PLT count, x 10^9^/L	<140	43 (11.0)	30 (14.4)	73 (12.2)
	140–440	334 (85.4)	169 (80.9)	503 (83.3)
	>440	14 (3.6)	6 (2.9)	20 (3.3)
	UNK	0	4 (1.9)	4 (0.7)
Alb, g/dL	<3.5	35 (9.0)	19 (9.1)	54 (9.0)
	≥3.5	356 (91.0)	186 (89.0)	542 (90.3)
	UNK	0	4 (1.9)	4 (0.7)
LDH, IU/L	≤ULN	270 (69.1)	91 (43.5)	361 (60.2)
	>ULN	96 (24.6)	94 (45.0)	190 (31.7)
	UNK	25 (6.4)	24 (11.5)	49 (8.2)

TABLE 1B. B Genomic Alterations of Patients with ≥1 Targetable Alterations by Pathway	No. of patients, Part A	No. of patients, Part B	Total no. of patients
	(*N* = 317)	(*N* = 157)	(*N* = 474)
PI3K/Akt/mTOR pathway	105 (33.1)	41 (26.1)	146 (30.8)
MAPK signaling	88 (27.7)	47 (29.9)	135 (28.4)
Tyrosine kinases	53 (16.7)	29 (15.5)	82 (17.2)
TP53	161 (50.8%)	69 (43.9%)	230 (48.5%)
Other (non-p53) tumor suppressor/apoptosis–associated genes	38 (12.0%)	10 (6.4%)	48 (10.1%)
Cell cycle–associated genes	128 (40.3)	37 (23.6)	165 (34.8)
Hormone pathway	7 (2.2)	1 (0.6)	8 (1.7)
DNA repair pathway	15 (4.7)	4 (2.5)	19 (4.0)

*Table 1A: Alb* albumin, *LDH* lactate dehydrogenase, *PLT* platelet, *PS* performance status, *UNK* unknown, *ULN* upper limit of normal.

*Table 1B: Akt* protein kinase B, *MAPK* mitogen-activated protein kinase, *mTOR* mammalian target of rapamycin, *PI3K* phosphoinositide 3-kinase, *TP53* tumor suppressor protein 53.

*Data cut-off 10/07/2021.

**Fig. 1:**
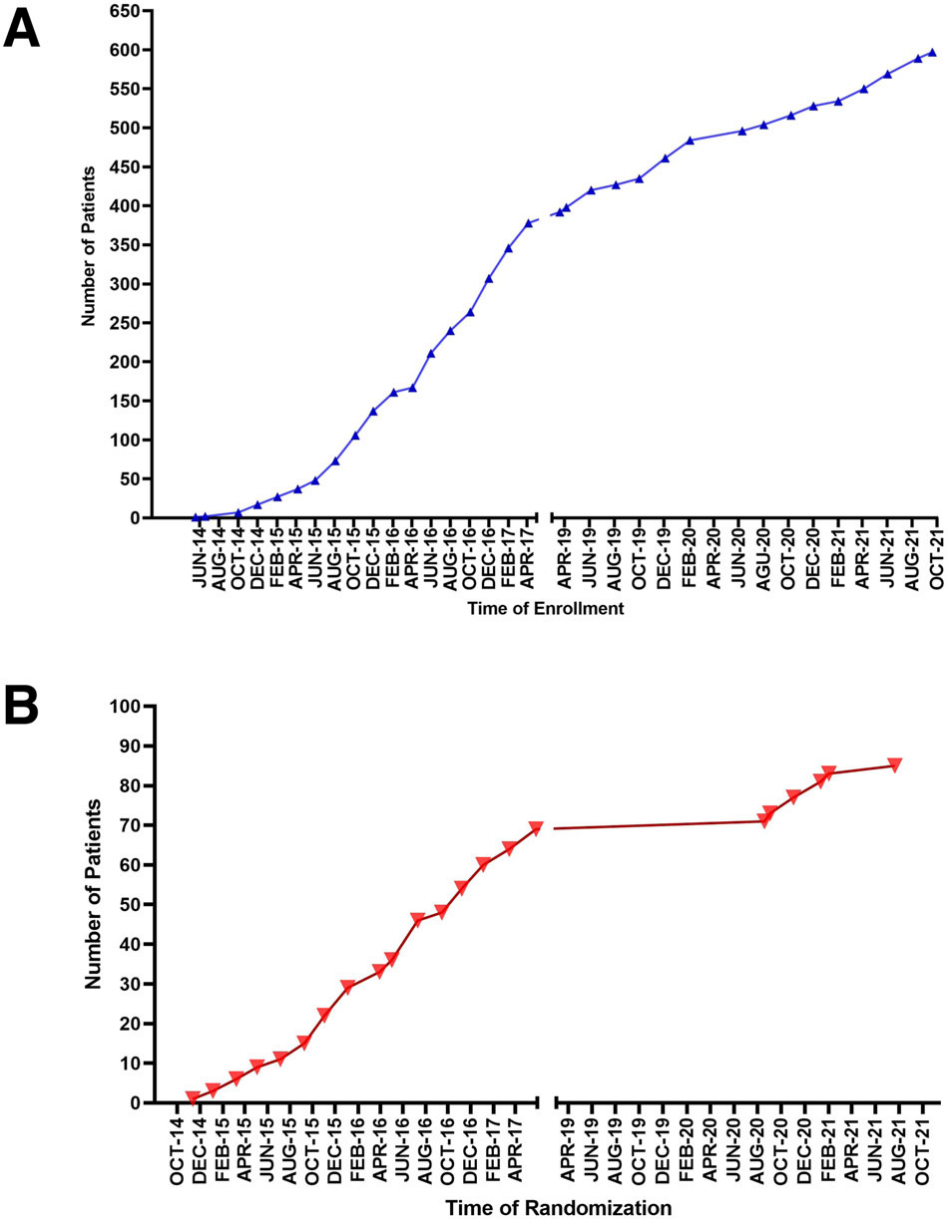
IMPACT2 enrollment and randomization over time. **A** Number of patients enrolled. **B** Number of patients randomized in Part A and Part B.

The genomic alterations by pathway are shown in Table 1B. Of 474 patients who had ≥1 targetable alteration, 230 (48.5%) patients had tumor protein P53 (*TP53*) alterations. Other commonly detected molecular alterations included cell cycle–associated genes (34.8%), phosphoinositide 3-kinase/AKT serine-threonine kinase/mammalian target of rapamycin (PI3K/AKT/mTOR) pathway alterations (30.8%), and mitogen-activated protein kinase (MAPK) signaling abnormalities (28.4%). The most common cancer types were gastrointestinal (*n* = 109, 18.2%) and head and neck (*n* = 88, 14.7%) (Table 2). Patient accrual by timing of enrollment and randomization are shown in Fig. 1.

**Table 2:** Tumor types.

Tumor type*	No. of patients, Part A (%)	No. of patients, Part B (%)	Total no. of patients (%)
	(*N* = 391)	(*N* = 209)	(*N* = 600)
GI, other (non-CRC)	65 (16.6)	43 (20.6)	108 (18.0)
Head and neck	68 (17.4)	20 (9.6)	88 (14.7)
CRC	35 (9.0)	38 (18.2)	73 (12.2)
Lung	48 (12.3)	12 (5.7)	60 (10)
Breast	31 (7.9)	21 (10)	52 (8.7)
Sarcoma	33 (8.4)	17 (8.1)	50 (8.3)
GYN, other (non-ovarian)	30 (7.7)	12 (5.7)	42 (7)
Prostate	15 (3.8)	16 (7.7)	31 (5.2)
Ovarian	20 (5.1)	6 (2.9)	26 (4.3)
GU, other (non-prostate)	23 (5.9)	0	23 (3.8)
Melanoma	8 (2.0)	12 (5.7)	20 (3.3)
Endocrine	12 (3.1)	4 (1.9)	16 (2.7)
Other cancers	0	7 (3.3)	7 (1.2)
CUP	3 (0.8)	1 (0.5)	4 (0.7)

*CRC* colorectal cancer, *CUP* cancer of unknown primary, *GI* gastrointestinal, *GU* genitourinary, *GYN* gynecologic.

*Data cut-off 10/07/2021.

### Part A

**Fig. 2:**
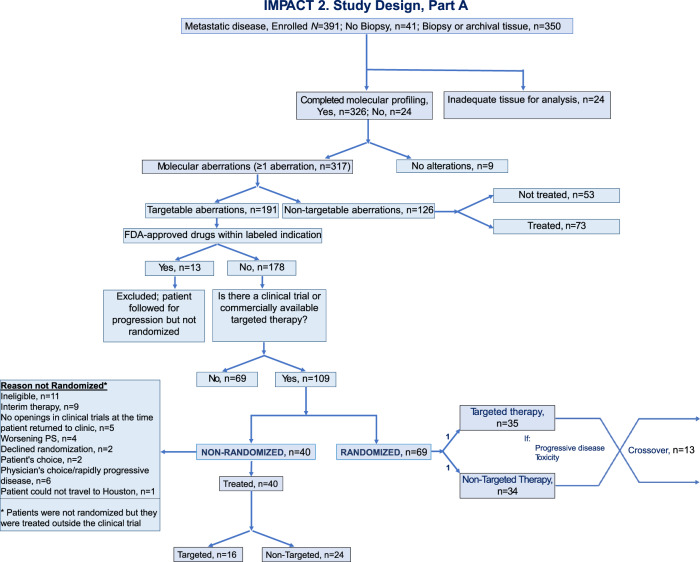
IMPACT2, Part A: Consort diagram.

From study activation on 5/13/14 until 4/30/17, 391 patients were enrolled on IMPACT2 and 326 completed tumor molecular profiling (Fig. 2). Overall, 317 (97.2%) of the 326 patients had ≥1 aberration (191 patients had targetable and 126 patients had non-targetable aberrations), and 69 (21.2%) patients were randomized (1:1 ratio, MTT: *n* = 35; non-MTT: *n* = 34). Table 3 summarizes the reasons patients were not randomized. Molecular pathway alterations of patients with ≥1 targetable alteration are listed in Table 1B.

**Table 3:** Status of randomization and reasons patients were not randomized.

Status of Randomization*	No. of patients, Part A (%)	No. of patients, Part B (%)
	(*N* = 391)	(*N* = 209)
Completed molecular profile	326	162
Pts. with molecular alterations	**317**	**157**
≥ 1 targetable alteration	191 (60.3)	148 (94.3)
Non-targetable alterations	126 (39.8)	9 (5.7)
FDA-approved drug for the indication		
Yes	13 (4.1)	3 (1.9)
No	178 (56.2)	145 (92.4)
**Part A**		
Clinical trial available		
No	69 (21.8)	
Yes	109 (34.4)	
Randomized	69 (21.8)	
Non-randomized	40 (12.6)	
Reason		
Ineligible	11 (3.5)	
Interim therapy	9 (2.8)	
Physician’s choice/rapidly progressive disease	6 (1.9)	
No clinical trial available	5 (1.6)	
Worsening PS	4 (1.3)	
Declined randomization	2 (0.6)	
Patient’s choice	2 (0.6)	
Patient could not travel to Houston	1 (0.3)	
**Part B**		
Offered randomization		
No		112
Yes		32
Accepted randomization		
Yes		16
No		16
Reason not offered randomization (*n* = 112)		
Patient and/or physician’s choice		46 (29.3)
Treatment was chosen prior to molecular profile report		26 (16.6)
Worsening PS/Death		20 (12.7)
No clinical trial available		10 (6.4)
Other reasons:		10 (6.4)
Ineligible for a clinical trial		5 (3.2)
Lost to follow-up		4 (2.6)
Complete response to prior immunotherapy		1 (0.6)

*PS*, performance status.

*Data cut-off 10/07/2021.

### Part B

**Fig. 3:**
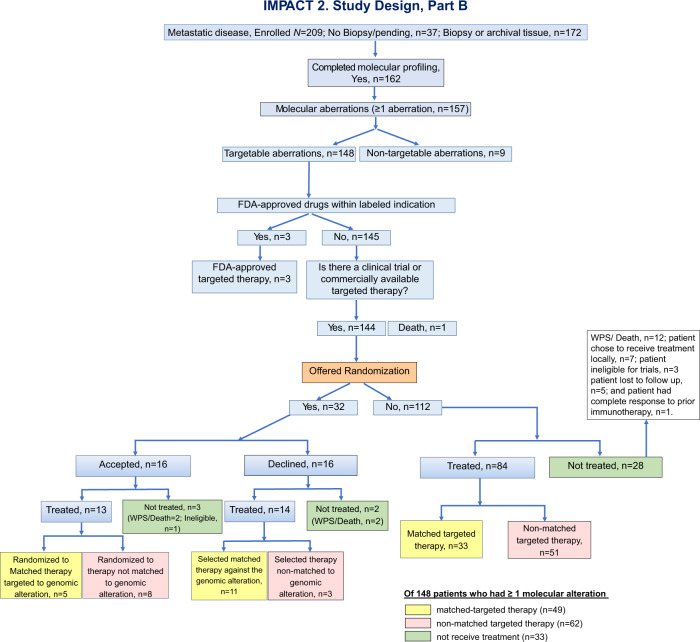
IMPACT2, Part B: consort diagram. Notably, patients who are randomized to matched targeted therapy and patients who select matched targeted therapy are to be analyzed together. Similarly, patients who are randomized to non-matched targeted therapy and patients who select non-matched targeted therapy are to be analyzed together.

From 3/20/2019 until 10/7/2021, 209 patients were enrolled on the study, and 162 completed molecular profiling. Of these 162 patients, 148 (91.3%) patients had at least 1 targetable alteration. Overall, 49 patients (33.1% of 148 patients with ≥1 targetable aberration) were assigned to MTT and 62 (41.9%) to non-MTT. Thirty-three (22.3%) patients did not receive treatment, and 16 (10.8%) patients were randomized (Fig. 3). Three of the 16 randomized patients were ineligible for treatment because of central nervous system metastases (*n* = 1) or worsening performance status (*n* = 2). The most commonly mutated genes were *TP53* (43.9%) and MAPK signaling–associated gene (29.9%) (Table 1B).

### Part B, randomization

Overall, 32 patients were offered randomization and 112 patients were not offered randomization. The reasons patients were not offered randomization are provided in Table 3. Of 16 patients who were offered and accepted randomization, 13 (81.2%) were treated. Five (38.5%) were randomized to MTT and 8 (61.5%) were randomized to non-MTT (Fig. 3). Sixteen patients declined randomization and 14 (87.5%) received treatment. Of these patients, 11 (78.6%) selected MTT and 3 (21.4%) selected non-MTT. Overall, 84 patients who were not offered randomization received treatment (MTT, *n* = 33; non-MTT, *n* = 51).

### Part B, non-treated patients

**Fig. 4:**
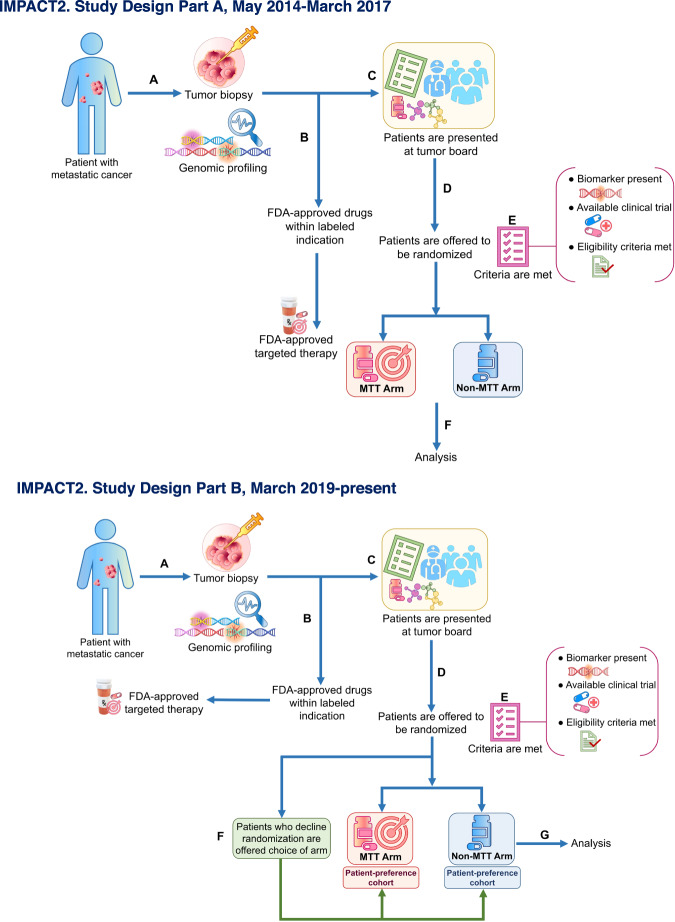
IMPACT2 study algorithm. **IMPACT2. Study design Part A, May 2014-March 2017** **A** Patients with metastatic cancer undergo a tumor biopsy and genomic profiling. **B** If patients have targetable molecular aberrations and FDA-approved drugs within labeled indication are available, the patients will receive FDA-approved targeted therapy. **C** If patients have targetable molecular aberrations and there are no FDA-approved drugs within labeled indication, the patients are considered for commercially available targeted therapy or clinical trial. Patients are presented at tumor board. **D** Patients are offered randomization to one of two arms: MTT or non-MTT **E** Criteria (biomarker present, available clinical trial, eligibility criteria met) are met. **F** Analysis. **IMPACT2. Study design Part B, March 2019-present.** **A** Patients with metastatic cancer undergo a tumor biopsy and genomic profiling. **B** If patients have targetable molecular aberrations and FDA-approved drugs within labeled indication are available, the patients will receive FDA-approved targeted therapy. **C** If patients have targetable molecular aberrations and there are no FDA-approved drugs within labeled indication, the patients are considered for commercially available targeted therapy or clinical trial. Patients are presented at tumor board. **D** Patients are offered randomization between two arms: MTT or non-MTT **E** Criteria (biomarker present, available clinical trial, eligibility criteria met) are met. **F** In March 2019, we amended the trial to include a “patient-preference” cohort for each arm. Patients who decline randomization are offered their choice of arm. **G** The primary analysis will use both randomized and patient-preference cohorts based on a Bayesian hierarchical model that “borrows” from the patient-preference cohorts to the extent to which its PFS agrees with that in the randomization cohort.

Thirty-three patients with ≥1 molecular alteration were not treated owing to the following reasons: worsening performance status or death (*n* = 16); patient chose to receive treatment locally (*n* = 7); patient ineligible for trials, (*n* = 4: lab abnormalities, *n* = 1; history of hepatitis, *n* = 1; newly diagnosed brain metastasis, *n* = 1, tuberculosis-positive on screening, *n* = 1); patient lost to follow-up (*n* = 5); and patient had complete response to prior immunotherapy (*n* = 1) (Fig. 4).

### Challenges associated with randomization

The key barriers to randomizing patients with actionable alterations varied in the two parts of the study. In Part A, of 317 patients with tumor molecular alterations, 21.8% of patients were randomized. The remaining patients were not randomized for the following reasons: absence of targetable alterations (39.8%), availability of FDA-approved drugs for the indication (4.1%), unavailability of clinical trial (21.8%), and various other reasons (12.6%, as listed in Table 3).

In Part B, of 157 patients with molecular alterations, 32 (20.4%) were offered randomization: 16 agreed to be randomized and 16 rejected randomization and selected the treatment arm. The remaining patients were not offered randomization for the following reasons: absence of targetable alterations (5.7%), availability of FDA-approved drugs for the indication (1.9%), treatment chosen prior to reports of molecular profiling (16.6%), worsening performance status or death (12.7%), patient’s choice and/or physician’s choice (29.3%), unavailability of clinical trials (6.4%), and other reasons (6.4%) (Table 3, Fig. 3, and Supplemental Information). Challenges for randomization are summarized in Table 4. Examples of non-matched treatments are listed in Supplemental Table 1.

**Table 4:** Challenges for randomization.

Challenges	Details
Time required for molecular testing	• Prolonged time interval from patient enrollment to availability of results (~3–4 weeks).
	• Need for immediate intervening therapy.
Ineligibility for clinical trials	• Targeted therapy already administered as standard of care.
	• Worsening PS/death.
	• Laboratory abnormalities, organ insufficiency.
	• Newly diagnosed brain metastases requiring immediate treatment; for most protocols, brain metastases should be stable for ≥4 weeks after completion of treatment.
	• Prior infections (HBV, HCV, HIV) that may compromise patients’ participation in clinical trials due to concern about virus reactivation.
	• Delayed response from prior immunotherapy.
Resources/finances	• Prolonged time to obtain financial clearance for participation in clinical trials
	• Logistic challenges requiring resources to comply with protocol procedures.
	• Travel, a particularly challenging requirement during the initial phase of the COVID-19 pandemic.
Patient selection	• With the revised design many patients selected to be treated with targeted therapy (Supplemental Fig. 1)

### Annotation and actionability of molecular alterations without available targeted therapy or selected trial option

While a significant proportion of patients who underwent molecular testing in the IMPACT2 study were reported to have actionable alterations that were matched to FDA-approved drugs for the indication, off-label drugs, or targeted agents in selected trials, many alterations of theoretically targetable pathways had no treatment options. Of note, in our previous paper reporting preliminary results of IMPACT2 we included an unbiased pathway enrichment analysis of applicable genes (utilizing the Panther pathway database) in patients with advanced cancer and their associations with overall survival^6^. However, not all genes in a targetable pathway have been validated to confer sensitivity to available drugs with a sufficient level of evidence to be considered actionable or for which there is an open trial selecting for them. Furthermore, although a trial may be selecting for a particular actionable biomarker, enrollment of the patient on a specific trial arm or cohort can be highly influenced by various factors including real-time cohort open/close status, slot availability, and other eligibility criteria of the cohort such as performance status, number of previous treatments, type of previous treatments, or the presence of brain metastasis or leptomeningeal disease^7^.

A comprehensive list of molecular pathways and alterations found in the study participants’ tumors for which targeted therapy or a selected trial option was not available is shown in Supplemental Table 2. Notably, certain genes are only actionable for specific mutations. For instance, *TP53* mutations in solid tumors are mostly only actionable for the Y220C mutation (in a TP53 Y220C reactivator clinical trial). *CTNNB1* is actionable for S37F, S45F/P, and T41A mutations (in a clinical trial). Some alterations were functionally significant, and at the time of treatment it was determined there were no matched treatment options available, but such options became available at a later time. In addition, some genes were considered “actionable” because they had diagnostic or prognostic implications. Other genes were considered biologically relevant but not actionable (e.g. loss-of-function mutation in an oncogene) based on their function or pathway relationship, but they had no specific predictive association with matched therapies and, therefore, such therapies could not be offered to patients. According to the protocol design, variants of unknown significance (VUS) were not acted upon.

## Discussion

IMPACT2 was initiated in 2014 as the largest randomized trial in precision oncology with adaptive design across tumor types. The adaptive design of IMPACT2 enables the study to incorporate additional molecular alterations and treatments as they become available. Molecular profiling using fresh tumor biopsy specimens, annotation of genomic alterations/immune marker results, treatment selection based on tumor board and multidisciplinary conference decisions, and patient randomization are essential study procedures. Patients are treated on selected clinical trials with MTT or non-MTT (or FDA-approved drugs) and are monitored for response and toxicity. Since we initiated the IMPACT program at MD Anderson Cancer Center^3,8,9^ (Supplemental Fig. 1), and the IMPACT2 study, several challenges to randomization have been encountered (Table 4).

Randomization challenges have changed over the 7-year period of the IMPACT2 study. In the first part of the study, 13 patients had FDA-approved drugs for their indication and the availability of targeted therapies was limited. Some of the molecular alterations that were considered not targetable when genomic results were used for treatment selection became targetable later, as new targeted agents entered clinical trials. Another challenge associated with conducting the IMPACT2 study and enrolling patients is the requirement to exhaust standard-of-care treatment. Starting with patient 47, patients with metastatic disease had to exhaust all established standard-of-care therapies, a criterion not required for similar studies such as NCI-MATCH (National Cancer Institute Molecular Analysis for Therapy Choice). In the latter study, patients must have progressed following at least one line of standard systemic therapy (clinicaltrials.gov, NCT02465060). As a result of the revised criterion, heavily pre-treated patients whose disease had progressed on multiple therapies frequently had worsening performance status and did not meet the criteria for randomization. Tumor evolution associated with progressive disease and accumulating genomic alterations may also hinder the efficacy of treatment. In addition to evolving eligibility criteria during the study period of IMPACT2, several changes occurred: (a) the use of molecular profiling and targeted therapy increased, (b) many targeted therapies were approved by the FDA; (c) the coverage of the gene panels increased; and (d) immunotherapy became available.

Guidelines for the interpretation and clinical significance of sequence variants have been developed by the Association for Molecular Pathology, American Society of Clinical Oncology, College of American Pathologists^10^, and European Society for Medical Oncology^11^. A universally applicable variant reporting system has been proposed by other investigators^12^. In a comparison of several level-of-evidence scales, we found that although each scale has its own nomenclature and nuance, their underlying concepts are overlapping^13^. Variant interpretation changes over time, and therefore, while we have maintained our core framework standardizing variant interpretation, published data influencing the actionability of variants are constantly evolving. Taking into consideration these guidelines, combined with our published framework, in IMPACT2 every patient was reviewed for actionability of their molecular alterations at a Tumor Board meeting. Additionally, clinical trial selection criteria for biomarkers and cohort availability are highly dynamic and ultimately impact treatment options.

Challenges associated with molecular testing included the prolonged time period from patient enrollment to availability of molecular profiling results (3–4 weeks), which often necessitated intervening therapy to prevent deterioration of performance status or organ dysfunction owing to advanced metastatic cancer. Several patients in Part B were not offered randomization because their molecular analysis results were available only after treatment selection. Additionally, after the amendment to allow patients to choose not to undergo randomization, some patients selected MTT or non-MTT without randomization (Table 3).

Regarding eligibility for clinical trials, some patients who had ≥ 1 targetable molecular alteration were identified to have FDA-approved drugs for their tumor type and therefore were excluded from randomization according to the study design. Additionally, patients were not randomized because they were ineligible for trials and/or randomization (Table 3).

Challenges associated with treatment after randomization include insurance approval for off-label drugs and treatment on clinical trials and logistical issues. Nine patients in Part A of IMPACT2 did not receive the assigned treatment on the randomized arm because their insurance did not approve the associated cost.

Drug-related barriers to randomization are associated with the unavailability of MTT against key driver biomarkers. Overall, 133 patients (27.2% of 488 patients who completed molecular profiling) had non-targetable alterations at the time of the analysis and therefore were not considered for randomization. Other randomized (French SHIVA^14^ and NCI’s MPACT [Molecular Profiling-based Assignment of Cancer Therapy]^15^) and non-randomized (WINTHER^16^ and TAPUR [Targeted Agent and Profiling Utilization Registry], NCT02693535^17^) trials evaluating molecular profiling and precision oncology and their results are summarized in Supplemental Information.

The strengths of IMPACT2 include an adaptive design that enables patient randomization despite the FDA approval of novel targeted therapies, expanding identifiable biomarkers, and the abundance of investigational drugs. Fresh tumor biopsies for identification of gene/immune biomarkers and a multidisciplinary approach were implemented to optimize treatment selection using a large set of clinical trials.

The difference in the absence of targetable alterations between patients in Parts A and B of the study (40% vs. 6%) is attributed to the dramatic decrease in non-targetable alterations owing to rapidly evolving data from basic and translational research. This not only improved our understanding of genetic actionability but also led to the discovery and development of novel targeted therapies and expanded clinical trials that use biomarkers to select patients. The gene panels were also expanded in Part B to increase coverage and sensitivity to detect alterations.

The rates of enrollment and randomization were lower in the second part of the study compared to the first part. Several factors contributed to these changes, which are evident by the decreased slope in the enrollment line in Fig. 2. First, the transition from Part A to Part B required a protocol amendment and change in sponsor. By the time the study was reactivated, 2 years after the completion of Part A, many targeted therapies had been approved by the FDA, resulting in fewer patients being referred to participate in the study. Second, giving patients the choice between randomization and treatment selection resulted in fewer patients being randomized. Third, time from enrollment to tumor biopsy has increased from 1–2 days in Part A to at least 7 days in Part B because of various factors that include prolonged time to schedule a tumor biopsy. Also, the change in the eligibility criteria requiring that patients had exhausted standard therapy, and the additional time required for completion of molecular testing, made “bridging” therapy necessary for the vast majority of patients who were enrolled in Part B. By the time patients complete bridging therapy, the ability to act on the molecular alterations is limited by various factors, including loss of eligibility for clinical trials owing to worsening performance status and organ function and logistical issues.

The IMPACT2 study has several limitations. First, as with other clinical trials across tumor types, it involves a large variety of alterations and tumors associated with complex biology and tumor plasticity. Second, tumors may harbor multiple molecular alterations that cannot be effectively targeted by the available therapies, except for checkpoint inhibitors targeting tumors bearing a high tumor molecular burden. Other unidentified mechanisms may contribute to carcinogenesis. As the study spans a few years, some investigational agents that were considered non-MTT at the time of treatment assignment were later proven to be MTT (e.g., immunotherapeutic agents targeting high tumor molecular burden). Third, the prolonged time period from the tumor biopsy to the profiling report (median, 19 days) often made bridging therapy necessary to avoid disease progression and delayed MTT. Accelerating the performance of tumor biopsies, genomic sequencing, and the annotation of molecular alterations and shortening the time to obtain financial clearance for insurance coverage of selected therapies will offer patients more timely access to clinical trials and MTT. The key challenge associated with randomization (as in similar studies) is differences in the characteristics of patients in the randomized arms, even after stratification.

To overcome the complexity of tumor biology, particularly in the advanced metastatic setting of solid tumors, drug combinations and novel strategies should be developed. These strategies should be available earlier in the course of the disease. In patients with complex molecular abnormalities, single-agent experimental treatment may offer only a transient benefit, if any^18^. The identified molecular alteration(s) may not represent the driver biomarker(s), or the molecular landscape may differ between the tumor of origin and metastatic sites^19^—a limitation that may be partially addressed using cell-free DNA analysis^20,21^. In the vast majority of investigational agents, preclinical antitumor activity does not translate into clinical benefit in humans. In IMPACT2, each patient is presented at a multidisciplinary conference that integrates the patient’s molecular profiles, including key drivers of the tumor, with available clinical trials of single investigational agents or combinations to optimize treatment planning and selection. Cell-free DNA analysis is undergoing validation and will soon be integrated to assist treatment planning.

The promise of precision oncology has yet to be fully realized and several gaps still exist. Thus far, MTT has been implemented without the benefit of randomized data, an approach that also applied to immunotherapy. Molecular profiling at the time of diagnosis and longitudinal molecular testing will optimize treatment selection during the early stages of disease progression. In IMPACT2, tumor molecular profiles of patients are utilized for treatment selection as the next step in management or at the time of disease progression after bridging therapy.

In conclusion, randomized controlled trials have been considered the gold standard for drug development, but the execution of randomized trials in precision oncology in the advanced metastatic setting is complicated. We have described the challenges associated with randomization from our experience of conducting the IMPACT2 study, a large precision oncology trial in patients with diverse solid tumor types. The adaptive design of IMPACT2 enables patient randomization despite the continual FDA approval of targeted therapies, the evolving tumor biomarker landscape, and the plethora of investigational drugs. Outcomes for randomized patients are awaited.

## Methods

The database of IMPACT2 and patient records were reviewed to determine presenting features, patient status on protocol, and reasons patients were not randomized.

### Eligibility

The eligibility criteria for IMPACT2 include patients with metastatic cancer who have had unlimited lines of prior therapy and have an Eastern Cooperative Oncology Group (ECOG) performance status of 0 or 1. Patients must have measurable disease, biopsy-accessible tumor, and normal organ and marrow function (absolute neutrophil count ≥ 1000/µl; platelets ≥ 100,000/µl), adequate hepatic function (bilirubin ≤ 1.5 x upper limit of normal [ULN]; alanine transaminase ≤ 2.5 x ULN), and serum creatinine clearance ≥50 ml/min (Cockcroft-Gault formula). Additional eligibility criteria include no brain metastasis or treated, stable, and/or asymptomatic brain metastasis for ≥4 weeks (off steroids ≥ 2 weeks). If patients have had previous malignancies, they must be disease-free ≥3 years. Patients are excluded from this study if they have had prior chemotherapy, surgery, or radiotherapy within 3 weeks of initiating study treatment. Additional exclusion criteria include severe cardiac conditions, peripheral neuropathy ≥grade 2, and concurrent severe and/or uncontrolled medical disease that could compromise participation in the study. Patients are also excluded if they have refractory nausea, vomiting, and/or chronic gastrointestinal diseases or have undergone a significant bowel resection that would preclude adequate absorption (for oral therapy only).

### Study Design

In Part A of IMPACT2 (Fig. 4), patients with metastatic cancer underwent tumor biopsies for genomic profiling. Genomic alterations were reviewed for interpretation and clinical significance by the MD Anderson Precision Oncology Decision Support (PODS) team. Characterization of the genomic alterations was driven by the level of evidence, and their actionability was determined using data from our own framework, as previously published^22,23^. The variant annotation in our database is updated every 6 months for variants of unknown significance and yearly for variants of established clinical significance. Results were discussed at an IMPACT2-specific molecular tumor board meeting and a multidisciplinary treatment planning conference. If a patient had a genomic abnormality targeted by an FDA-approved drug for the specific tumor type, the patient received the FDA-approved drug. Investigational treatment options were also discussed. In June 2015, the eligibility criteria were revised, and starting with patient number 47, prior to randomization, patients had to exhaust all established standard-of-care therapies, or physicians had to determine that such established therapy was not sufficiently efficacious, or patients had to decline to receive standard-of-care therapy. In March 2019, the trial was amended to include Part B, a “patient-preference” cohort, for each arm, as previously described^24–26^. Specifically, in the “patient-preference” cohort, patients eligible for randomization are offered randomization to MTT vs. non-MTT, and those who decline to be randomized can select their preferred cohort. The rationale for this revised design was based on the crucial question of whether the results of randomized controlled trials are influenced by preference effects. Patients’ preferences gained attention in response to concerns regarding the ethics of randomization and maintenance of the equipoise principle (therapeutic uncertainty). Consequently, a mathematical construct was developed for additive and two-way interactions between preference, guessed and actual treatments, and treatment outcomes^24–26^. Briefly, patients who decline randomization are now offered their choice of the two trial arms (MTT or non-MTT; NCT02152254) (Fig. 4). The data are to be analyzed with “intention-to-treat.” The primary analysis will use both randomized and patient-preference cohorts based on a Bayesian hierarchical model^27–29^ that “borrows” from the patient-preference cohort to the extent to which its PFS agrees with that in the randomization cohort. The randomization rate is 1:1, and randomization stratification includes 8 groups: epidermal growth factor receptor (*EGFR*), B-Raf proto-oncogene (*BRAF*), phosphoinositide 3-kinase (*PI3K*), phosphatase and tensin homolog (*PTEN*), MET proto-oncogene (*MET*), kirsten rat sarcoma viral oncogene homolog (*KRAS*), human epidermal growth factor receptor 2 (*ERBB2*), and “other” (i.e., other genomic alterations and immune markers including tumor mutational burden [TMB], microsatellite instability [MSI] status, and programmed death-ligand 1 [PD-L1] to select immunotherapy).

### Biopsies and molecular profiling using next-generation sequencing

Tumor tissue is obtained via core biopsy performed by interventional radiology. If tissue is not accessible, bronchoscopy, upper endoscopy, or colonoscopy are performed to access tissue, as determined by the treating physician.

Tissue samples are assessed by a pathologist to ensure ≥ 20% tumor cellularity prior to sending them for genomic analysis. For patients who have undergone tumor resection or biopsy within 1 year prior to enrollment on the trial and have received up to two lines of anticancer therapy in the interim, the remaining available tissue can be used for molecular profiling if the pathologist confirms that it is adequate for molecular analysis.

Molecular testing was performed in CLIA-certified laboratories. In the first part of the study (Part A, May 2014-March 2017), molecular profiling was performed by Foundation Medicine, as previously described^6^. Briefly, DNA isolated from formalin-fixed, paraffin-embedded (FFPE) tumor tissue samples from patients was sequenced using FoundationOne CDx™, a comprehensive next-generation sequencing–based in vitro diagnostic device designed to detect substitutions, insertion and deletion alterations (indels), copy number alterations, and rearrangements in 315 genes. DNA sequencing was performed using the HiSeq-2000 instrument (Illumina), with 49 × 49 paired-end reads. Starting in November 2016, tumor tissue samples were also assessed for immunotherapy targets that included TMB, MSI status, and PD-L1 protein expression.

In the second part of the study (Part B, March 2019-present), genomic testing is being performed at Tempus using the Tempus xT assay^30–33^, which included a 595-gene targeted sequencing panel from April 2019 to October 2019 and a 648-gene panel starting in November 2019. FFPE samples and a matched normal saliva or blood sample are sequenced to detect somatic single-nucleotide variants, indels, copy number variants, gene rearrangements, and MSI. Copy number variants are derived from a proprietary tumor/normal-matched analysis using the R package CNATools. The library preparation is performed using the KAPA Hyper prep kit followed by target capture with custom-designed Roche probes. Alignment and mapping to the GRCh37 reference genome are performed using NovoAlign and the Burrows–Wheeler Aligner. Variant Call Format files are annotated using the R package SnpEff, with detected variants categorized as either somatic or germline. TMB, MSI status, and PD-L1 protein expression by immunohistochemical analysis were also assessed.

As part of the Tempus xT assay, a whole-transcriptome panel is also being performed and used for expanded detection of gene rearrangements, immune infiltration, and gene expression for immune- and targeted therapy–related genes. The library preparation is performed using the KAPA Hyper prep kit followed by exome capture using IDT xGen Lockdown® Probes, Exome Research Panel v1.0. Alignment and mapping to the reference genome GRCh38 are performed using STAR alignment. Transcript abundances are expressed as transcripts per million and assessed by Kallisto. Expression calls are made using Feature Counts.

### Definition of matched targeted therapy

An “actionable” mutation is defined as a genomic alteration in an individual’s tumor that is targetable with an available therapeutic agent or is the target of a novel therapy in development.

Targeted therapies are drugs or other substances that inhibit cancer by interfering with specific molecules (i.e., molecular targets) involved in the growth, progression, and/or spread of cancer. Targeted cancer therapies are sometimes called “molecularly targeted drugs,” “molecularly targeted therapies,” or “precision medicines.” FDA-approved targeted therapies for the treatment of patients with specific cancer types include hormone therapies, signal transduction inhibitors, gene expression modulators, apoptosis inducers, angiogenesis inhibitors, immunotherapies, and toxin-delivery molecules. Other targeted therapies are being investigated in clinical trials or in preclinical development^2^. Recently, the term targeted therapy began to include immunotherapeutics. High (>10 mutations/megabase [mut/mb]) TMB, deficient mismatch repair (dMMR)/MSI, PD-L1 expression by immunohistochemical analysis, and other evolving immune markers are used to assign matched targeted immunotherapy to patients.

### Tumor board meetings

Tumor board members, including molecular biologists with expertise in precision oncology, medical oncologists, the study statistician, radiologists, and research scientists, meet weekly. Research nurses and study and data coordinators also participate in the meetings. Tumor molecular alterations are evaluated on the basis of the level of evidence that is available at the time results of molecular profiling are discussed. Currently, somatic mutations are categorized as somatic, potentially actionable; somatic, biologically relevant; or germline, pathogenic/likely pathogenic. Variants of unknown significance are discussed but they are not taken into consideration in treatment assignment. Immunotherapy markers that include TMB, MSI (stable, equivocal, or high), PD-L1 expression, tumor proportion score, combined positive score, and DNA mismatch repair protein expression are also reviewed, and they are taken into consideration in treatment assignment. RNA profile-expression details (no treatment implications) are presented. Clinical trials under consideration may include targeted therapy, immunotherapy, and/or novel agents.

### Ethics approval and consent to participate

The protocol was approved by the Institutional Review Board at The University of Texas MD Anderson Cancer Center on May 19, 2014 (Part A), and the amended protocol (Part B) was approved on March 18, 2019. The assigned protocol number is PA12-1161. The study was conducted in accordance with the Declaration of Helsinki and the International Conference on Harmonization of Good Clinical Practice guidelines. All the study participants provided written informed consent before enrollment stating that they were aware of the investigational nature of the study.

### Reporting summary

Further information on research design is available in the Nature Research Reporting Summary linked to this article.

## Supplementary information

Supplemental material

REPORTING SUMMARY

## Data Availability

Data are available upon reasonable request. The datasets used and/or analyzed during the current study are available from the corresponding author upon reasonable request and approval from study sponsor and institution according to available guidelines at the time of request. The data generated and analyzed for Part A of the study^6^ were previously published in the following metadata record: 10.6084/m9.figshare.1364342022. The two European Genome-phenome Archive (EGA) accession codes (data are subject to controlled-access) are: https://identifiers.org/ega.dataset:EGAD00001006887 (dataset ID) and https://identifiers.org/ega.study:EGAS00001004964 (study ID). The data generated for Part B of the study have been collected. The data of Part B and the overall study will be analyzed upon its completion, according to the IRB requirements for this randomized study. Sharing the data of Part B will require IRB-approved collaboration, a specified Data Usage Agreement, and will be for non-commercial use only. For data inquiries, please contact the corresponding author Dr. Apostolia-Maria Tsimberidou, email address: atsimber@mdanderson.org. The owner of the data of the study is The University of Texas, MD Anderson Cancer Center.
