# Supplementary material for: Challenges and opportunities associated with the MD Anderson IMPACT2 randomized study in precision oncology
Source: NPJ Precis Oncol. 2022 Oct 27;6:78. doi: 10.1038/s41698-022-00317-0 (PMC9612600; doi:10.1038/s41698-022-00317-0)
Supplement: Supplementary file 1 — Supplemental material [file 41698_2022_317_MOESM1_ESM.pdf]

SUPPLEMENTAL FILE

Supplemental notes

Introduction

When IMPACT2 study was initiated, there were several challenges to the implementation of precision oncology, including but not limited to the following: (A) Tumor biopsy for molecular profiling was not the standard of care (SOC) as it was for certain hematologic malignancies, despite accumulating evidence showing that the genomic plasticity of cancer cells over time affects drug sensitivity [1]. (B) Comprehensive molecular testing using next-generation sequencing in a CLIA-certified laboratory was not extensively used. (C) Bioinformatic analysis and annotation of the clinical significance of genomic alterations were suboptimal. (D) MTT agents were not available for many driver molecular alterations and access to available MTTs was limited owing to the lack of patient insurance coverage and clinical trials. (E) Evidence-based selection of drugs was not standardized. Additional challenges include the prolonged period of time required for molecular profiling (10-15 days) and the suboptimal use of "N-of-1" databases to enable application of knowledge gained from previously treated patients.

Other clinical trials evaluating molecular profiling and precision oncology

Other phase II, randomized trials evaluating molecular profiling and precision oncology include the French SHIVA study [2] and NCI's MPACT (Molecular Profiling-based Assignment of Cancer Therapy) study [3]. In SHIVA, the first randomized trial in precision oncology, no difference in PFS between patients treated with MTT and those treated according to physician choice was reported (HR: 0.88, 95% CI .65-1.19, *p*=0.41) [2]. SHIVA had several limitations [2,4], including suboptimal [5] treatment predefined by a treatment algorithm in the MTT arm compared with treatment selected by treating physicians in the control group, increasing the probability of selection bias. In NCI-

MPACT, patients were randomized (2:1 ratio) to receive either (1) a study regimen identified to target the aberrant pathway found in their tumor (veliparib with temozolomide; adavosertib with carboplatin [DNA repair pathway], everolimus [PI3K pathway], or trametinib [RAS/RAF/MEK pathway]) or (2) one of the remaining three regimens not targeting that pathway [3]. Of 49 randomized and treated patients, only patients treated with trametinib had a partial response. ORR and PFS comparisons between the two arms were not performed owing to the high dropout rate [3]. The limitations of the study included but were not limited to study design (i.e., randomization between one matched targeted therapy based on pathway alteration versus one of three other non-targeting agents resulting in suboptimal treatment assignment) and lack of real-time sharing of molecular profiling results with the treating physicians. In IMPACT2, genomic results are shared in real time with physicians and patients. Tumor board and multidisciplinary approaches are used to optimize treatment selection, eliminating bias. More importantly, the adaptive design of IMPACT2 enables patient treatment even as molecular profiling and available treatments continue to expand.

We also participated in WINTHER, another non-randomized, international study across tumor types in precision oncology. Patients (n=107) were assigned to MTT combinations on the basis of genomic (Arm A) or transcriptomic (Arm B) alterations [6]. Better outcomes were noted in patients who were well-matched compared with those who were poorly matched to therapy, and transcriptomic analysis complemented genomic analysis, increasing the proportion of patients who received MTT [6]. After we started IMPACT2, several large, non-randomized, phase II studies were initiated for patients with advanced malignancies across tumor types, including the NCI-MATCH (National Cancer Institute-Molecular Analysis for Therapy Choice) and TAPUR (Targeted Agent and Profiling Utilization Registry) trials. NCI-MATCH (NCT02465060), which launched in 2015, evaluates MTT selected on the basis of specific tumor alterations [7]. The investigators reported rates of participant enrollment [8,9], and results from some subprotocols [10-15],

demonstrating the feasibility of molecular profiling and treatment assignment [16]. As of January 8, 2022, 9 arms were open, 9 were suspended, and 21 arms were closed [17]. Positive results were reported for three arms (nivolumab for non-colorectal cancers with mismatch repair–deficient; capivasertib for diverse tumor types with AKT1 E17K mutations; and dabrafenib and trametinib for diverse tumor types harboring BRAFV600E mutations) [10,18,19], negative for four [20-23], and for the remaining arms the accrual is ongoing [17]. TAPUR is a multi-basket, phase II precision oncology trial that integrates tumor genomic profiling using tumor biopsy and/or liquid biopsy to match patients with diverse advanced cancers to off-label use of targeted anticancer agents (NCT02693535) [24]. As of September 13, 2021, positive results were reported for 11 cohorts [25-35], negative for three [36-38], and results for the remaining cohorts are pending [39].

Supplemental Table 1. Examples of non-matched therapy

Type of treatment	Examples
Chemotherapy-containing regimens	Eribulin and trastuzumab<br>Topotecan and paclitaxel<br>FOLFIRI<br>Carboplatin plus paclitaxel<br>Nab-paclitaxel<br>Pemetrexate and bevacizumab<br>Gemcitabine<br>Gemcitabine and docetaxel<br>Paclitaxel
Novel agents	HDAC6 inhibitor[40]<br>Alkylating agent<br>Wee1 inhibitor<br>IDH2 inhibitor<br>STAT3 inhibitor<br>TROP2 ADC
Anti-VEGF/Multi-kinase inhibitors *	Lenvatinib<br>Pazopanib<br>Investigational
Immunotherapy	Nivolumab plus Ipilimumab
Other, standard-of-care	Palbociclib (CDK4/6 inhibitor) *<br>Olaparib (PARP inhibitor) *<br>Everolimus and Exemestane *
Investigational	Multikinase inhibitor plus PD1 inhibitor<br>Locoregional therapy plus FOLFOX<br>T-Cell therapy<br>CTLA4 and PD1 inhibitors

* These agents were not matched to patients' tumor molecular profile; they were selected by the treating physicians as standard-of-care.

Abbreviations: ADC: antibody-drug conjugate; CDK4: cyclin-dependent kinase 4; CDK6: cyclin-dependent kinase 6; CTLA4: cytotoxic T-lymphocyte-associated protein 4; FOLFIRI: folinic acid, fluorouracil, irinotecan; FOLFOX: folinic acid, fluorouracil, and oxaliplatin; HDAC6: histone deacetylase 6; IDH2: isocitrate dehydrogenase type 2; PARP: poly (ADP-ribose) polymerase; PD1: programmed cell death protein 1; STAT3: signal transducer and activator of transcription 3; TROP2: trophoblast cell surface antigen 2; VEGF: vascular endothelial growth factor

Supplemental Table 2. Study patients' molecular pathways and alterations without available targeted therapy or a selected trial option

Molecular pathway	Molecular alterations
p53 pathway feedback loops 2	TP53: p.F113fs frameshift – LOF; TP53: p.R248W missense variant – LOF; TP53: p.L194R missense variant – LOF; TP53: p.R175H missense variant – LOF; TP53: p.P278L missense variant – LOF; TP53: p.R248W missense variant; TP53: p.R248Q missense variant – LOF ; TP53: p.R213* stop gain – LOF; TP53: p.H179fs frameshift – LOF; TP53: p.Y163C missense variant; TP53: copy number loss; TP53: p.P191fs frameshift – LOF; TP53: p.M169fs frameshift – LOF; TP53: p.R306* stop gain – LOF; TP53: p.RP249SS missense variant – LOF; TP53: p.H214R missense variant – LOF; TP53: p.D48fs frameshift – LOF; TP53: p.E271* stop gain – LOF; TP53: p.V157F missense variant; TP53: p.Q144* stop gain – LOF; TP53: splice site 920-15_993+40del129; TP53: p.R282W missense variant ; TP53: p. C275Y missense variant – LOF; CTNNB1: p.A39_K49del inframe deletion – GOF; CTNNB1: p.G34V missense variant – GOF
Angiogenesis	CRKL: Copy number loss; APC: p.Y1075* stop gain – LOF; APC: p.P1424fs frameshift – LOF; APC: p.T1556fs frameshift – LOF; APC: p.L1488fs frameshift – LOF; APC: p.R232* stop gain – LOF; APC: p.R1450* stop gain – LOF; APC: p.R564* stop gain – LOF; APC: p.Q1378* stop gain – LOF; APC: p.K1165* Stop gain – LOF; APC: p.E1309fs frameshift – LOF; APC: p.S1389fs frameshift – LOF; APC: p.E443* stop gain – LOF; APC: p.Y1376* stop gain – LOF ; APC: p.R499* stop gain – LOF; NOTCH2: copy number loss; FRS2: copy number gain; CTNNB1: p.A39_K49del inframe deletion – GOF; CTNNB1: p.G34V missense variant – GOF; VEGFA: copy number gain
FGF signaling pathway	FRS2: copy number gain
p53 pathway	TP53: p.F113fs frameshift – LOF; TP53: p.R248W missense variant – LOF; TP53: p.L194R missense variant – LOF; TP53: p.R175H missense variant – LOF; TP53: p.P278L missense variant – LOF; TP53: p.R248W missense variant; TP53: p.R248Q missense variant – LOF ; TP53: p.R213* stop gain – LOF; TP53: p.H179fs Frameshift – LOF; TP53: p.Y163C missense variant; TP53: copy number loss; TP53: p.P191fs frameshift – LOF; TP53: p.M169fs frameshift – LOF; TP53: p.R306* stop gain – LOF; TP53: p.RP249SS missense variant – LOF; TP53: p.H214R missense variant – LOF; TP53: p.D48fs frameshift – LOF; TP53: p.E271* stop gain – LOF ; TP53: p.V157F missense variant; TP53: p.Q144* stop gain – LOF; TP53: splice site 920-15_993+40del129; TP53: p.R282W missense variant ; TP53: p. C275Y missense-LOF; PTEN: p.R233* stop gain – LOF; CREBBP: p.E1012* stop gain – LOF
Hypoxia response via HIF activation	CREBBP: p.E1012* stop gain – LOF
PDF signaling pathway	JAK1: p.L431fs frameshift – LOF; JAK1: p.S683fs frameshift – LOF; JAK1: copy number loss.

Endothelin signaling pathway	GNAS: p.R201H missense variant – GOF
CCKR signaling map	PTPN11: p.G503E missense variant – GOF; PTPN11: p.T507K missense variant – GOF activating; PTPN11: c.1380-1G>T splice region variant – LOF; PTPN11: p.G462* stop gain – LOF; CTNNB1: p.A39_K49del inframe deletion – GOF; CDH1: p.G577fs frameshift – LOF
Interleukin signaling pathway	CDKN1B: p.W76fs frameshift – LOF
Hedgehog signaling pathway	CREBBP: p.E1012* stop gain – LOF
JAK/STAT signaling pathway	JAK1: p.L431fs frameshift – LOF; JAK1: p.S683fs frameshift – LOF; JAK1: copy number loss
p53 pathway feedback loops 2: confer resistance to drugs, non-targetable	The following mutations confer resistance to CDK4/6 inhibitors: RB1: c.1498+2T>G splice region variant – LOF; RB1: p.R552* stop gain – LOF; RB1: p.V654fs frameshift – LOF; RB1: c.1215+1G>A splice region variant – LOF; RB1: P. Y173fs frameshift – LOF

Abbreviations: GOF: gain-of-function; LOF: lost-of-function

REFERENCES

1 Sequist, L. V. *et al.* Genotypic and histological evolution of lung cancers acquiring resistance to EGFR inhibitors. *Sci. Transl. Med.* **3**, 75ra26, doi:10.1126/scitranslmed.3002003 (2011).

2 Le Tourneau, C. *et al.* Molecularly targeted therapy based on tumour molecular profiling versus conventional therapy for advanced cancer (SHIVA): a multicentre, open-label, proof-of-concept, randomised, controlled phase 2 trial. *Lancet Oncol.* **16**, 1324-1334, doi:10.1016/S1470-2045(15)00188-6 (2015).

3 Chen, A. P. *et al.* Molecular Profiling-Based Assignment of Cancer Therapy (NCI-MPACT): A Randomized Multicenter Phase II Trial. *JCO Precis Oncol* **5**, doi:10.1200/PO.20.00372 (2021).

4 Tsimberidou, A. M. & Kurzrock, R. Precision medicine: lessons learned from the SHIVA trial. *Lancet Oncol.* **16**, e579-580, doi:10.1016/S1470-2045(15)00397-6 (2015).

5 Janku, F. *et al.* Assessing PIK3CA and PTEN in early-phase trials with PI3K/AKT/mTOR inhibitors. *Cell Rep.* **6**, 377-387, doi:10.1016/j.celrep.2013.12.035 (2014).

6 Rodon, J. *et al.* Genomic and transcriptomic profiling expands precision cancer medicine: the WINTHER trial. *Nat. Med.* **25**, 751-758, doi:10.1038/s41591-019-0424-4 (2019).

7 ECOG-ACRIN. *NCI-MATCH precision medicine cancer trial*, <https://ecog-acrin.org/nci-match-eay131> (2020).

8 Flaherty, K. T. *et al.* The Molecular Analysis for Therapy Choice (NCI-MATCH) Trial: Lessons for Genomic Trial Design. *J Natl Cancer Inst* **112**, 1021-1029, doi:10.1093/jnci/djz245 (2020).

9 Chen, A. P. *et al.* Abstract PL03-01: NCI-MATCH: A new paradigm in the era of genomic oncology. *Molecular cancer therapeutics* **17**, PL03-01-PL03-01, doi:10.1158/1535-7163.Targ-17-pl03-01 (2018).

10 Azad, N. S. *et al.* Nivolumab Is Effective in Mismatch Repair-Deficient Noncolorectal Cancers: Results From Arm Z1D-A Subprotocol of the NCI-MATCH (EAY131) Study. *J. Clin. Oncol.* **38**, 214-222, doi:10.1200/JCO.19.00818 (2020).

11 Kalinsky, K. *et al.* in *EORTC-NCI-AACR.*

12 Jhaveri, K. L. *et al.* Ado-trastuzumab emtansine (T-DM1) in patients with HER2-amplified tumors excluding breast and gastric/gastroesophageal junction (GEJ) adenocarcinomas: results from the NCI-MATCH trial (EAY131) subprotocol Q. *Annals of Oncology* **30**, 1821-1830, doi:10.1093/annonc/mdz291 (2019).

13 Krop, I. *et al.* Results from molecular analysis for therapy choice (MATCH) arm I: Taselisib for PIK3CA-mutated tumors. *Journal of clinical oncology : official journal of the American Society of Clinical Oncology* (2018).

14 Kummar, S. *et al.* Abstract CT138: NCI-MATCH EAY131 -Z1I: Phase II study of AZD1775, a wee-1 kinase inhibitor, in patients with tumors containing BRCA1 and BRCA2 mutations. *Cancer Research* **79**, CT138-CT138, doi:10.1158/1538-7445.Am2019-ct138 (2019).

15 Chae, Y. K. *et al.* Molecular analysis for therapy choice (MATCH) arm W: Phase II study of AZD4547 in patients with tumors with aberrations in the FGFR pathway. *Journal of Clinical Oncology* **36**, 2503, doi:10.1200/JCO.2018.36.15_suppl.2503 (2018).

16 Flaherty, K. T. *et al.* The Molecular Analysis for Therapy Choice (NCI-MATCH) Trial: Lessons for Genomic Trial Design. *J. Natl. Cancer Inst.* **112**, 1021-1029, doi:10.1093/jnci/djz245 (2020).

17 *NCI-MATCH / EAY131*, <https://ecog-acrin.org/trials/nci-match-eay131> (

18 Kalinsky, K. *et al.* Effect of Capivasertib in Patients With an AKT1 E17K-Mutated Tumor: NCI-MATCH Subprotocol EAY131-Y Nonrandomized Trial. *JAMA Oncol* **7**, 271-278, doi:10.1001/jamaoncol.2020.6741 (2021).

19 Salama, A. K. S. *et al.* Dabrafenib and Trametinib in Patients With Tumors With BRAF(V600E) Mutations: Results of the NCI-MATCH Trial Subprotocol H. *J. Clin. Oncol.* **38**, 3895-3904, doi:10.1200/JCO.20.00762 (2020).

20 Jhaveri, K. L. *et al.* Ado-trastuzumab emtansine (T-DM1) in patients with HER2-amplified tumors excluding breast and gastric/gastroesophageal junction (GEJ) adenocarcinomas: results from the NCI-MATCH trial (EAY131) subprotocol Q. *Ann. Oncol.* **30**, 1821-1830, doi:10.1093/annonc/mdz291 (2019).

21 Johnson, D. B. *et al.* Trametinib Activity in Patients with Solid Tumors and Lymphomas Harboring BRAF Non-V600 Mutations or Fusions: Results from NCI-MATCH (EAY131). *Clin Cancer Res* **26**, 1812-1819, doi:10.1158/1078-0432.CCR-19-3443 (2020).

22 Chae, Y. K. *et al.* Phase II Study of AZD4547 in Patients With Tumors Harboring Aberrations in the FGFR Pathway: Results From the NCI-MATCH Trial (EAY131) Subprotocol W. *J. Clin. Oncol.* **38**, 2407-2417, doi:10.1200/JCO.19.02630 (2020).

23 Cleary, J. M. *et al.* Differential Outcomes in Codon 12/13 and Codon 61 NRAS-Mutated Cancers in the Phase II NCI-MATCH Trial of Binimetinib in Patients with NRAS-Mutated Tumors. *Clin Cancer Res* **27**, 2996-3004, doi:10.1158/1078-0432.CCR-21-0066 (2021).

24 *Targeted Agent and Profiling Utilization Registry (TAPUR™) Study*, <https://www.tapur.org/> (2020).

25 Pisick, E. P. *et al.* Olaparib (O) in patients (pts) with prostate cancer with BRCA1/2 inactivating mutations: Results from the Targeted Agent and Profiling Utilization Registry (TAPUR) study. *J. Clin. Oncol.* **38**, 5567-5567, doi:10.1200/JCO.2020.38.15_suppl.5567 (2020).

26 Ahn, E. R. *et al.* Olaparib (O) in patients (pts) with pancreatic cancer with BRCA1/2 inactivating mutations: Results from the Targeted Agent and Profiling Utilization Registry (TAPUR) study. *J. Clin. Oncol.* **38**, 4637-4637, doi:10.1200/JCO.2020.38.15_suppl.4637 (2020).

27 Pisick, E. P. *et al.* Palbociclib (P) in patients (pts) with head and neck cancer (HNC) with CDKN2A loss or mutation: Results from the Targeted Agent and Profiling Utilization Registry (TAPUR) study. *J. Clin. Oncol.* **39**, 6043-6043, doi:10.1200/JCO.2021.39.15_suppl.6043 (2021).

28 Ahn, E. R. *et al.* Palbociclib in Patients With Non–Small-Cell Lung Cancer With CDKN2A Alterations: Results From the Targeted Agent and Profiling Utilization Registry Study. *JCO Precision Oncology*, 757-766, doi:10.1200/PO.20.00037 (2020).

29 Schuetze, S. *et al.* Palbociclib (P) in patients (pts) with soft tissue sarcoma (STS) with CDK4 amplification: Results from the Targeted Agent and Profiling Utilization Registry (TAPUR) study. *J. Clin. Oncol.* **39**, 11565-11565, doi:10.1200/JCO.2021.39.15_suppl.11565 (2021).

30 Gupta, R. *et al.* Pertuzumab plus trastuzumab (P+T) in patients (Pts) with colorectal cancer (CRC) with ERBB2 amplification or overexpression: Results from the TAPUR Study. *Journal of Clinical Oncology* **38**, 132, doi:10.1200/JCO.2020.38.4_suppl.132 (2020).

31 Ali-Ahmad, H. M. *et al.* Pertuzumab plus trastuzumab (P+T) in patients (Pts) with uterine cancer (UC) with ERBB2 or ERBB3 amplification, overexpression or mutation: Results from the Targeted Agent and Profiling Utilization Registry (TAPUR) study. *J. Clin. Oncol.* **39**, 5508-5508, doi:10.1200/JCO.2021.39.15_suppl.5508 (2021).

32 Alva, A. S. *et al.* Pembrolizumab in Patients With Metastatic Breast Cancer With High Tumor Mutational Burden: Results From the Targeted Agent and Profiling Utilization Registry (TAPUR) Study. *J. Clin. Oncol.* **39**, 2443-2451, doi:10.1200/JCO.20.02923 (2021).

33 Meiri, E. *et al.* Pembrolizumab (P) in patients (Pts) with colorectal cancer (CRC) with high tumor mutational burden (HTMB): Results from the Targeted Agent and Profiling

Utilization Registry (TAPUR) Study. *J. Clin. Oncol.* **38**, 133-133, doi:10.1200/JCO.2020.38.4_suppl.133 (2020).

34 Calfa, C. *et al.* Abstract CT173: Sunitinib (S) in patients (pts) with metastatic breast cancer (mBC) with FGFR1 mutations or amplifications: Results from the Targeted Agent and Profiling Utilization Registry (TAPUR) Study. *Cancer Res.* **81**, CT173-CT173, doi:10.1158/1538-7445.Am2021-ct173 (2021).

35 Klute, K. *et al.* Cobimetinib plus vemurafenib (C+V) in patients (Pts) with colorectal cancer (CRC) with BRAF V600E mutations: Results from the TAPUR Study. *J. Clin. Oncol.* **38**, 122-122, doi:10.1200/JCO.2020.38.4_suppl.122 (2020).

36 Baghdadi, T. A. *et al.* Palbociclib in Patients With Pancreatic and Biliary Cancer With CDKN2A Alterations: Results From the Targeted Agent and Profiling Utilization Registry Study. *JCO Precision Oncology*, 1-8, doi:10.1200/po.19.00124 (2019).

37 Al Baghdadi, T. *et al.* Sunitinib in Patients with Metastatic Colorectal Cancer (mCRC) with FLT-3 Amplification: Results from the Targeted Agent and Profiling Utilization Registry (TAPUR) Study. *Target. Oncol.* **15**, 743-750, doi:10.1007/s11523-020-00752-8 (2020).

38 Fisher, J. G. *et al.* Cetuximab in Patients with Breast Cancer, Non-Small Cell Lung Cancer, and Ovarian Cancer Without KRAS, NRAS, or BRAF Mutations: Results from the Targeted Agent and Profiling Utilization Registry (TAPUR) Study. *Target. Oncol.* **15**, 733-741, doi:10.1007/s11523-020-00753-7 (2020).

39 *Summary of Cohort Activity in the TAPUR Study*, <https://www.asco.org/research-data/tapur-study/study-results> (

40 Tsimberidou, A. M. *et al.* Preclinical Development and First-in-Human Study of KA2507, a Selective and Potent Inhibitor of Histone Deacetylase 6, for Patients with Refractory Solid Tumors. *Clin Cancer Res* **27**, 3584-3594, doi:10.1158/1078-0432.CCR-21-0238 (2021).

41 Tsimberidou, A. M. *et al.* Personalized medicine in a phase I clinical trials program: the MD Anderson Cancer Center initiative. *Clin Cancer Res* **18**, 6373-6383, doi:10.1158/1078-0432.CCR-12-1627 (2012).

42 Tsimberidou, A. M. *et al.* Personalized medicine for patients with advanced cancer in the phase I program at MD Anderson: validation and landmark analyses. *Clin Cancer Res* **20**, 4827-4836, doi:10.1158/1078-0432.CCR-14-0603 (2014).

43 Tsimberidou, A. M. *et al.* Initiative for Molecular Profiling and Advanced Cancer Therapy (IMPACT): An MD Anderson Precision Medicine Study. *JCO Precis Oncol*, Epub 2017, doi:10.1200/PO.17.00002 (2017).

44 Tsimberidou, A. M. *et al.* Long-term overall survival and prognostic score predicting survival: the IMPACT study in precision medicine. *J Hematol Oncol* **12**, 145, doi:10.1186/s13045-019-0835-1 (2019).

SUPPLEMENTAL FIGURE LEGEND

Supplemental Figure 1. Timeline chart of IMPACT studies

Supplemental Figure 1

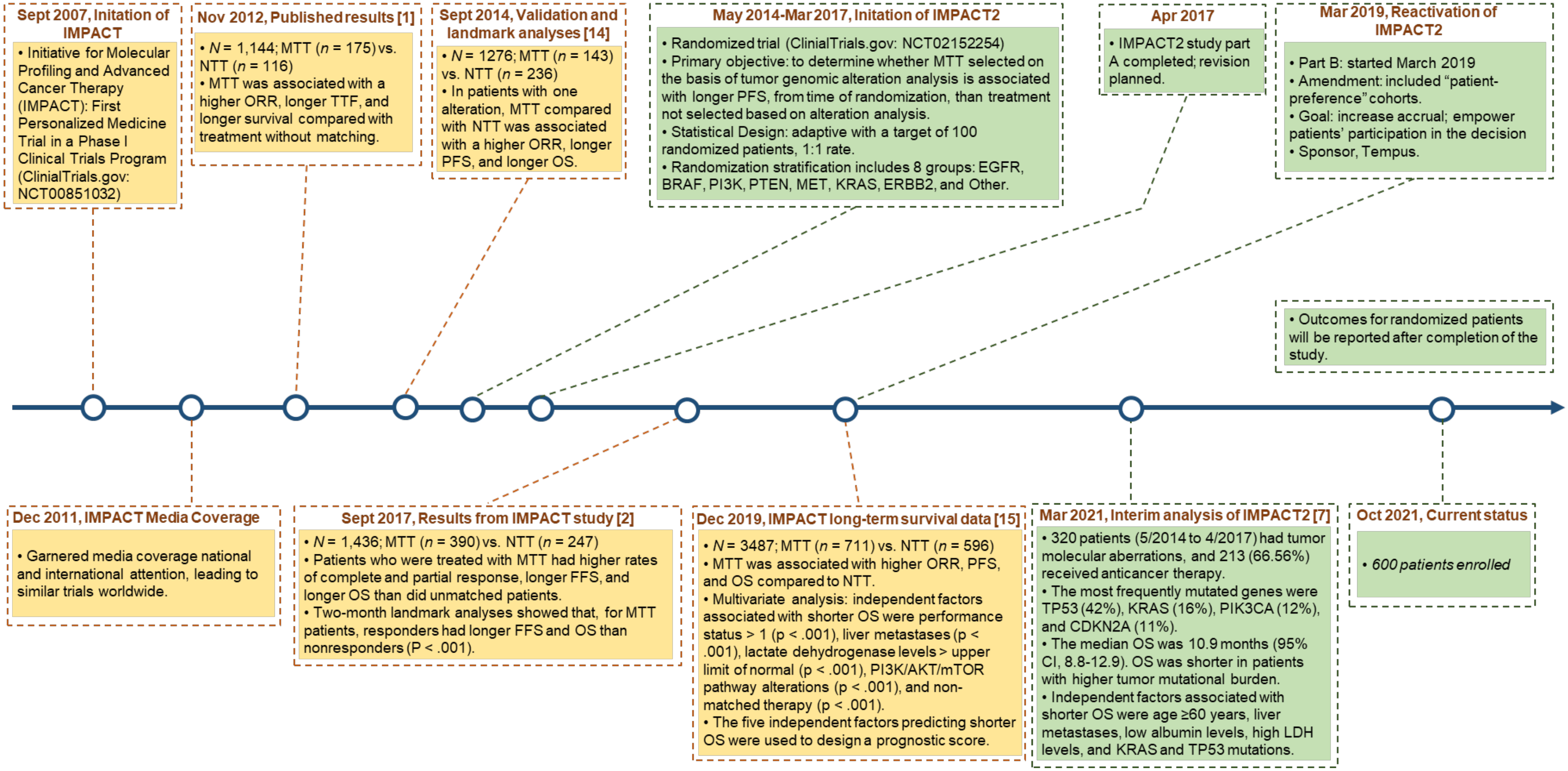

IMPACT, first part [41]				IMPACT, second part, validation analysis, 2014 [42]			IMPACT, third part, 2017 [43]			IMPACT, long-term analysis [44]		
N = 1144				*N* = 1276			*N* = 1436			*N* = 3487		
	MTT	NTT	*P*-value	MTT	NTT	*P*-value	MTT	NTT	*P*-value	MTT	NTT	*P*-value
	175	116		143	236		390	247		711	596	
ORR, %	27	5	<0.0001	12	5	0.0001	11	5	0.0099	16.4	5.4	0.0001
TTF, median, months	5.2	2.2	<0.0001	3.9	2.2	0.001	FFS, 3.4	2.9	0.0015	PFS, 4.0	2.8	0.0001
OS, median, months	13.4	9.0	0.017	11.4	8.6	0.04	8.4	7.3	0.041	9.3	7.3	0.0001

Abbreviations: FFS, failure-free survival; MTT, matched targeted therapy; NTT, non-targeted therapy; ORR, overall response rate; OS, overall survival; PFS, progression-free survival; TTF, time to treatment failure
