# Supplementary material for: Challenges and opportunities associated with the MD Anderson IMPACT2 randomized study in precision oncology
Source: NPJ Precis Oncol. 2022 Oct 27;6:78. doi: 10.1038/s41698-022-00317-0 (PMC9612600; doi:10.1038/s41698-022-00317-0)
Supplement: Supplementary file 2 — REPORTING SUMMARY [file 41698_2022_317_MOESM2_ESM.pdf]

nature portfolio

Corresponding author(s): Apostolia-Maria Tsimberidou
Last updated by author(s): 8/2/2022

Reporting Summary

Nature Portfolio wishes to improve the reproducibility of the work that we publish. This form provides structure for consistency and transparency in reporting. For further information on Nature Portfolio policies, see our Editorial Policies and the Editorial Policy Checklist.

Statistics

For all statistical analyses, confirm that the following items are present in the figure legend, table legend, main text, or Methods section.

n/a	Confirmed	
☐	☒	The exact sample size (*n*) for each experimental group/condition, given as a discrete number and unit of measurement
☒	☐	A statement on whether measurements were taken from distinct samples or whether the same sample was measured repeatedly
☒	☐	The statistical test(s) used AND whether they are one- or two-sided<br>*Only common tests should be described solely by name; describe more complex techniques in the Methods section.*
☒	☐	A description of all covariates tested
☒	☐	A description of any assumptions or corrections, such as tests of normality and adjustment for multiple comparisons
☒	☐	A full description of the statistical parameters including central tendency (e.g. means) or other basic estimates (e.g. regression coefficient) AND variation (e.g. standard deviation) or associated estimates of uncertainty (e.g. confidence intervals)
☒	☐	For null hypothesis testing, the test statistic (e.g. *F*, *t*, *r*) with confidence intervals, effect sizes, degrees of freedom and *P* value noted<br>*Give P values as exact values whenever suitable.*
☒	☐	For Bayesian analysis, information on the choice of priors and Markov chain Monte Carlo settings
☒	☐	For hierarchical and complex designs, identification of the appropriate level for tests and full reporting of outcomes
☒	☐	Estimates of effect sizes (e.g. Cohen's *d*, Pearson's *r*), indicating how they were calculated

Our web collection on statistics for biologists contains articles on many of the points above.

Software and code

Policy information about availability of computer code

Data collection	n/a
Data analysis	n/a

For manuscripts utilizing custom algorithms or software that are central to the research but not yet described in published literature, software must be made available to editors and reviewers. We strongly encourage code deposition in a community repository (e.g. GitHub). See the Nature Portfolio guidelines for submitting code & software for further information.

Data

Policy information about availability of data

All manuscripts must include a data availability statement. This statement should provide the following information, where applicable:
- Accession codes, unique identifiers, or web links for publicly available datasets
- A description of any restrictions on data availability
- For clinical datasets or third party data, please ensure that the statement adheres to our policy

Data are available upon reasonable request. The datasets used and/or analyzed during the current study are available from the corresponding author upon reasonable request and approval from study sponsor and institution according to available guidelines at the time of request.

Human research participants

Policy information about studies involving human research participants and Sex and Gender in Research.

Reporting on sex and gender	Sex and/or gender was determined based on patients' self-reporting.
Population characteristics	N/A
Recruitment	The participants were enrolled in MD Anderson Cancer Center. All consecutive patients who were referred to the phase 1 program were screened and enrolled on the trial regardless of their age, sex/gender, socioeconomic status, or any other factors that could introduce bias.
Ethics oversight	Institutional Review Board at The University of Texas MD Anderson Cancer Center

Note that full information on the approval of the study protocol must also be provided in the manuscript.

Field-specific reporting

Please select the one below that is the best fit for your research. If you are not sure, read the appropriate sections before making your selection.

☒ Life sciences ☐ Behavioural & social sciences ☐ Ecological, evolutionary & environmental sciences

For a reference copy of the document with all sections, see nature.com/documents/nr-reporting-summary-flat.pdf

Life sciences study design

All studies must disclose on these points even when the disclosure is negative.

Sample size	Total patient number: 600
Data exclusions	no data exclusion
Replication	n/a
Randomization	This is a randomized trial. Patient were randomly assigned to matched-targeted therapy or to non-matched targeted therapy. The trial includes a patient preference cohort (please see methods for details).
Blinding	not blinded

Reporting for specific materials, systems and methods

We require information from authors about some types of materials, experimental systems and methods used in many studies. Here, indicate whether each material, system or method listed is relevant to your study. If you are not sure if a list item applies to your research, read the appropriate section before selecting a response.

Materials & experimental systems

n/a	Involved in the study	
☒	☐	Antibodies
☒	☐	Eukaryotic cell lines
☒	☐	Palaeontology and archaeology
☒	☐	Animals and other organisms
☐	☒	Clinical data
☒	☐	Dual use research of concern

Methods

n/a	Involved in the study	
☒	☐	ChIP-seq
☒	☐	Flow cytometry
☒	☐	MRI-based neuroimaging

Clinical data

Policy information about clinical studies

All manuscripts should comply with the ICMJE guidelines for publication of clinical research and a completed CONSORT checklist must be included with all submissions.

Clinical trial registration	NCT02152254

Study protocol	The trial is ongoing and it is available upon request. The study information can be found: https://clinicaltrials.gov/ct2/show/NCT02152254.
Data collection	The trial is ongoing in MD Anderson Cancer Center. Data reported on patients enrolled from 5/19/2014 to 10/7/2021.
Outcomes	The endpoint of the study is progression-free survival (PFS), i.e., to determine whether patients treated on the basis of tumor genomic alterations have longer PFS than those whose treatment is not selected on the basis of genomic alterations. The study is ongoing and the endpoint will be reported at the end of the trial.
